# Analysis of Protein Degradation and Umami Peptide Release Patterns in Stewed Chicken Based on Proteomics Combined with Peptidomics Approach

**DOI:** 10.3390/foods14142497

**Published:** 2025-07-16

**Authors:** Lei Cai, Qiuyu Zhu, Lili Zhang, Ruiyi Zheng, Baoguo Sun, Yuyu Zhang

**Affiliations:** 1 Key Laboratory of Geriatric Nutrition and Health, Beijing Technology and Business University, Ministry of Education, Beijing 100048, China; cailei_1219@163.com (L.C.); zhuqiuyu0821@163.com (Q.Z.); yolo17807640109@163.com (R.Z.); sunbg@btbu.edu.cn (B.S.); 2 Food Laboratory of Zhongyuan, Beijing Technology and Business University, Beijing 100048, China; 3 Key Laboratory of Flavor Science of China General Chamber of Commerce, Beijing Technology and Business University, Beijing 100048, China; 4 School of Food and Health, Beijing Technology and Business University, Beijing 100048, China; 5 Beijing Life Science Academy, Beijing 102209, China

**Keywords:** chicken, protein, umami peptide, release pattern, peptidomics

## Abstract

Proteomics combined with peptidomics approaches were used to analyze the protein degradation and the release pattern of umami peptides in stewed chicken. The results showed that a total of 422 proteins were identified, of which 273 proteins consistently existed in samples stewed for 0–5 h. Myosin heavy chain exhibited the highest abundance (26.29–30.26%) throughout the stewing process. The proportion of proteins under 20 kDa increased progressively with the duration of stewing and reached 61% at 4–5 h of stewing. A total of 8018 peptides were detected in the soup samples, and 2323 umami peptides were identified using the prediction platforms iUmami-SCM, UMPred-FRL, Umami_YYDS, and TastePertides-DM. Umami peptides derived from titin (accession number A0A8V0ZZ81) were determined to be the most abundant, accounting for 24% of the total umami peptides, and Val534 and Lys33639 were the key N-terminal and C-terminal amino acids of these umami peptides. Abundance analysis showed that the umami peptides KK16 and SK18 ranked among the top 5 in the samples stewed for 0–5 h, and they were most abundant in the 3 h stewed samples. The results obtained will provide data support for promoting the industrialization of high-quality chicken soup products.

## 1. Introduction

Chicken is widely favored by consumers due to its palatable meat quality and high nutritional value. In China, chicken serves as the primary ingredient for countless regional delicacies with distinctive geographical characteristics, including Guangdong white-cut chicken, Sichuan saliva chicken, and Henan Daokou roast chicken. Chicken’s unique taste primarily stems from muscle-derived compounds. Currently, over 300 non-volatile components have been identified in chicken products, including more than 100 taste-active compounds. Among them, sugars, sugar alcohols, and sweet amino acids are recognized as major contributors to the sweetness in chicken [1,2,3,4,5,6]. Organic acids and sour peptides are identified as the main sources of sourness in chicken [7,8,9,10]. Metal cations and salty peptides are considered principal factors for saltiness in chicken [11,12]. Umami amino acids, 5′-nucleotides, and umami peptides are established as major umami determinants in chicken [13]. In addition, bitter amino acids, nucleotides, and bitter peptides are considered to be the key bitter components in chicken [14,15].

As analytical techniques continue to advance, more studies have focused on the contribution of peptides to chicken taste in addition to amino acids, nucleotides, and organic acids. Wang et al. analyzed the flavor peptides in Totole cooking chicken in broth and commercial chicken powder, and found that Totole cooking chicken in broth contained the highest content of small peptides with abundant umami and umami-enhancing peptides. Additionally, the dipeptide EE with prominent umami features was identified in Totole cooking chicken in suit [16]. Four peptides (LVQY, VHAHS, AQNSYPHA, AYRLVG) were identified in Beijing You chicken soup through high-performance liquid chromatography (HPLC) combined with matrix-assisted laser desorption ionization time-of-flight mass spectrometry (MALDI-TOF MS). These peptides were mainly composed of Gln, Ala, Ser, and other umami and sweet-associated amino acids, suggesting their significant contribution to the soup’s delicious taste [17]. In addition, 921 peptides were identified from Yanjin black-bone chicken using sensory-guided fractionation combined with liquid chromatography–tandem mass spectrometry (LC-MS/MS) by Yang et al., and 6 peptides (DTEEVEHGEE, EVHEEEVH, HEAEEVHEE, HEKLSEESEE, KVED, TPIPDLP) with umami flavor were further identified by amino acid composition analysis integrated with flavor activity prediction [18]. The increasing discovery of novel umami peptides in chicken substantially advances the analysis of its unique umami characteristics.

Umami peptides in chicken primarily originate from muscle protein degradation during cooking, alongside free peptides in muscle tissue. As an important method to improve the flavor quality and prolong the shelf life of meat, thermal processing changes the structure and composition of protein in muscle, which facilitates the dissolution of flavor compounds. Additionally, the peptides and amino acids produced by protein degradation serve as crucial flavor precursors, contributing significantly to the formation of meat’s unique flavor. The use of flavourzyme accelerates protein degradation during the fermentation process of grass carp, effectively shortening the fermentation cycle and enhancing the umami taste and flavor quality of the fermented products [19]. Studies on dry-cured ham processing indicate that oxidation, degradation, and thermal effects significantly alter protein surface hydrophobicity. This alteration plays a key role in generating characteristic flavor compounds, including aldehydes, ketones, esters, and sulfur compounds [20]. In a study of protein degradation and changes in non-volatile flavor compounds during steaming of *Portunus trituberculatus*, it was found that proteins were degraded to flavor precursors throughout the 0–25 min steaming process resulting in continuous changes in flavor characteristics, and the optimal taste was achieved by steaming for 10–15 min in conjunction with sensory evaluation and equivalent umami concentration (EUC) values [21]. Studies on the effects of cooking temperature and time on protein degradation and oxidation in chicken showed that maximal protein degradation and oxidation were detected at cooking temperatures of 80–90 °C and cooking times of 50–60 min [22]. With the intelligent development of cooking tools, meat processing has become more automated; however, the degradation characteristics of chicken proteins during actual cooking processes remain unclear.

To clarify the degradation characteristics of protein in chicken during the cooking process more concisely, the release patterns of umami peptides in chicken soup during thermal processing were analyzed. In this study, chicken meat was stewed using a nutrient-rich soup preparation protocol. The degradation characteristics of chicken protein were analyzed by a proteomics approach based on nano-liquid chromatography quadrupole time-of-flight mass spectrometry (Nano-LC-QTOF-MS). Additionally, a peptidomics analysis method combined with machine learning-based umami peptide prediction platforms was employed to elucidate changes in umami peptides during stewing. The results will support targeted improvements in the umami taste of chicken soup and advance the industrialization of high-quality chicken soup products.

## 2. Materials and Methods

### 2.1. Materials and Reagents

Chicken (90-week-old) was purchased from the Zhongcui Family Farm (Daiyue District, Taian, China). Ultrapure water was purchased from Watsons (Guangzhou, China). Formic acid (LC-MS grade, 98%) was purchased from J&K Scientific (Beijing, China). Acetonitrile (LC-MS grade, 99.9%) was purchased from Merck (Darmstadt, Germany). Sodium dodecyl sulfate (SDS), Urea, Tris (hydroxymethyl) aminomethane (Tris), Dithiothreitol (DTT), and Indole-3-acetic acid (IAA) were purchased from Bio-Rad (Hercules, CA, USA). KCl and HCl were purchased from Sinopharm Chemical Reagent Co., Ltd. (Shanghai, China). NH_4_HCO_3_ was purchased from Sigma-Aldrich (St. Louis, MO, USA).

### 2.2. Preparation of Chicken Soup

The chicken breast was separated (skin and adipose tissue removed) and cut into meat pieces with a side length of 2–3 cm, placed in a stew pot with a meat-to-water ratio of 1:1.5 (*w*/*w*), and heated in nutrient soup mode for 0–5 h. After cooling to room temperature, the meat pieces and soup were separated to obtain chicken meat and chicken soup samples, respectively.

### 2.3. Real-Time Temperature Detection in Stew Pot

In order to clarify the heating procedure in the stew pot and set the sampling time reasonably, a wireless temperature recorder (TDL-2 Rightec) was used to measure the temperature rise curve in the stew pot during the stewing process of chicken soup. When measuring the temperature, we completely submerged the probe in the chicken soup and avoided touching the wall of the stew pot.

### 2.4. Analysis of the Taste Characteristics

Following the previously established method [23], taste characteristics of chicken soup samples were analyzed using sensors (sour (CA0), bitter (C00), umami (AAE), salty (CT0), and sweet (GL1)) equipped with the taste sensing system SA-402B. Before the analysis experiment, the sensor and reference electrode were primed with internal solution (3.33 M HCl + saturated solution of AgCl) and immediately placed in the reference solution (3.33 M KCl solution) for 24 h (sensor GL1 needs to be immersed in a special immersion solution for sweet sensors). The GL1_test procedure was used for the determination of the sweetness of the sample (Table S1), and the Sample_Measurement (2steps_washing) procedure was used for the determination of sourness, saltiness, bitterness, and umami (Table S2). For each sample, 60 mL was aliquoted into electronic tongue-specific cups and loaded onto the sample tray. Sweetness was measured separately from other taste attributes. Each sample underwent quadruplicate measurements (5 times for sweetness), with the final three replicates retained for subsequent data processing.

### 2.5. Preparation of Label-Free Protein Analysis Sample

Chicken breast samples (*n* = 3, biological repeats) were ground into powder using liquid nitrogen. Proteins were extracted by SDT (4% (*w*/*v*) SDS, 100 mM Tris/HCl pH 7.6, 1 M DTT). The BCA method was used to quantify the proteins in the sample. Protein samples (150 μg) were reduced with 10 mM DTT (95 °C, 15 min) and cooled. After the sample had dropped to room temperature, 200 μL of UA buffer (8 M Urea, 150 mM Tris-HCl, pH 8.0) was added, the sample was transferred to a 10 kDa ultrafiltration centrifuge tube (14,000× *g*, 30 min), and the filtrate was discarded. An amount of 100 μL IAA buffer (50 mM IAA in UA buffer; 30 min, dark) was added. Urea was removed by repeated UA buffer washes, followed by equilibration with 50 mM NH_4_HCO_3_. Proteins were digested overnight (37 °C, 16 h) with trypsin (2 μg) in 40 μL NH_4_HCO_3_. Peptides were recovered by centrifugation, washed with additional NH_4_HCO_3_, desalted using C18 cartridges, and lyophilized.

### 2.6. Preparation of Peptidomics Analysis Sample

The sample (*n* = 3, biological repeats) was prepared according to Gallego et al.’s method, with minor modifications [24]. The chicken soup samples were cooled and filtered, and the filtrate was freeze-dried. An amount of 0.1 g of the freeze-dried chicken soup powder was added to 500 μL of 0.1% trifluoroacetic acid aqueous solution to reconstitute, and then centrifuged for 10 min (14,000× *g*). The supernatant was added to a 10 kDa ultrafiltration tube (PALL, OD010C35) and centrifuged for 10 min (13,500× g). The nanodrop 2000C UV-Vis spectrophotometer was used to quantitatively analyze the peptide content in the filtrate. At the same time, the peptide composition analysis of the filtrate was carried out after desalting by a C18 solid-phase extraction column. The steps are as follows: The extraction column was washed with acetonitrile and equilibrated with 0.1% trifluoroacetic acid solution for the desalting of ultrafiltration sample; the sample load was 1 mL, the eluent was 70% acetonitrile solution, the eluent was collected and freeze-dried, and the freeze-dried powder was reconstituted with 0.1% formic acid for mass spectrometry analysis.

### 2.7. LC-MS/MS Analysis

Nano-LC-QTOF-MS was used to identify the protein/peptide composition of pretreated samples. LC-MS/MS analyses were performed with three biological repeats, each representing an independently prepared sample batch.

Liquid phase conditions: Samples were separated using NanoLC Easy nLC-1200 (Thermo Scientific, Waltham, MA, USA). The mobile phase consisted of A (0.1% formic acid in water) and B (0.1% formic acid in 84% acetonitrile). The samples were enriched by a C18 loading column (100 μm × 2 cm, 5 μm) and separated by a C18 analytical column (75 μm × 10 cm, 3 μm); the flow rate, injection volume, and column temperature were set at 250 nL/min, 2 μL, and 50 °C, respectively. A gradient elution was carried out according to Table S3.

Mass spectrometry conditions: The chromatographically separated samples were analyzed using the Q-Exactive HF-X mass spectrometer. The positive ESI mode was used with an analysis time of 60 min. MS1 and MS2 conditions are shown in Table S4, with 10 fragmentation patterns (MS2 scans) acquired after each full scan.

### 2.8. Processing Mass Spectrometry Data

Mass spectrometry raw data files were processed using MaxQuant software (version 2.1.1.1) for protein/peptide identification. The relevant search parameters are listed in Table S5.

### 2.9. Statistical Analysis

The statistical analyses were performed using the statistical package SPSS 17.0 (SPSS Inc., Chicago, IL, USA). Statistical significance for protein/peptide content and peptide kinds was determined by one-way analysis of variance. The mean values were considered significantly different at *p* < 0.05. Statistical significance for the abundance of umami peptide was determined using the Kruskal–Wallis test (*, *p* < 0.05; **, *p* < 0.01; ***, *p* < 0.001).

## 3. Results and Discussion

### 3.1. Determination of Sampling Time

**Figure 1:**
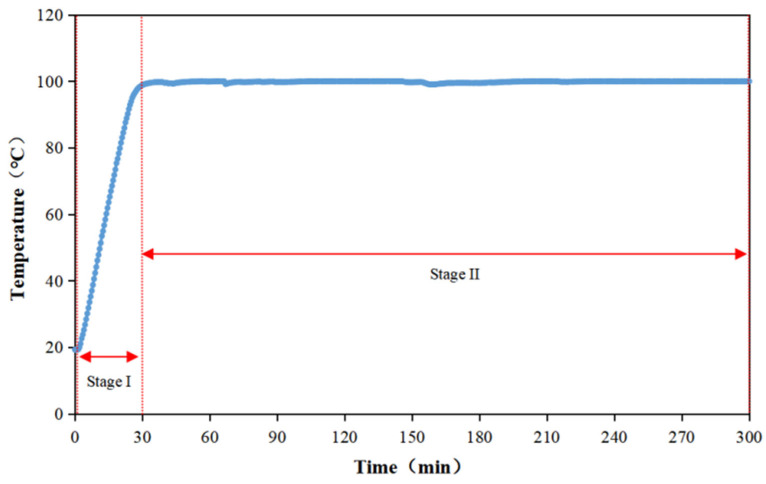
Warming curve of a stew pot in a nutrient soup mode.

The temperature profile within the stew pot during chicken soup preparation is shown in Figure 1. Two characteristic stages were observed in the stewing process: 0–0.5 h was the heating stage (Stage I), with a heating rate of 2.58 C/min; 0.5–5 h was the constant temperature stage (Stage II), with an average temperature of 99.81 ± 0.22 °C. Based on this thermal regime, seven sampling points were established at 0 h, 0.5 h, and hourly intervals from 1 h to 5 h.

### 3.2. Taste Characteristics Analysis of Chicken Soup

**Figure 2:**
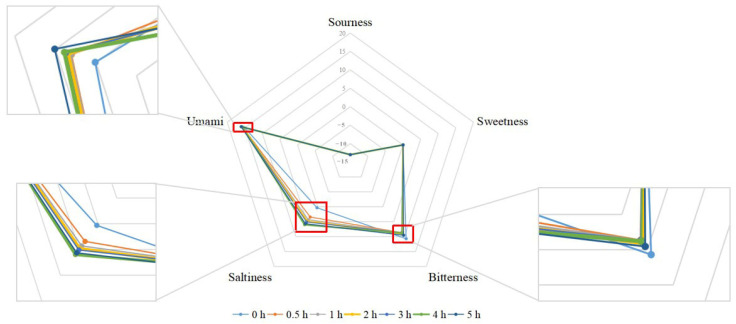
Analysis of taste characteristics of chicken soup at different heat treatment times.

The taste characteristics of the samples at different heat treatment times were analyzed using an electronic tongue, and the results are shown in Figure 2. As shown in Figure 2, the taste characteristics of chicken soup stewed for different times were mainly umami. The saltiness of the samples differed significantly and showed a positive correlation with the stewing time. Notably, the bitterness of the 0 h stewed samples was higher than that of samples stewed for longer durations. In a study by Duan et al., it was found that chicken soup was predominantly salty in the early stage of oral processing (0–3 s), and as time increased, saltiness perception intensity weakened and umami became the dominant taste [25], which was consistent with the results of the present work. The prominent umami characteristics of chicken soup are closely related to the abundant umami substances in it. These substances are continuously released at various stages of oral processing, activating umami receptors in the taste buds and stimulating downstream signals that are transmitted to the brain via nerves. This process triggers human perception of the umami taste in chicken soup. There was a positive correlation between the umami intensity and the saltiness intensity of the seven chicken soup samples, which may be related to the interaction between the taste perception of salty and umami substances [26].

### 3.3. Proteomics Characterization Analysis

During heat treatment, different muscle proteins undergo denaturation due to structural changes, resulting in different flavor qualities [27]. The analysis of total protein content in samples subjected to different heat treatment durations revealed an initial increase from 34.95 mg/mL to 62.68 mg/mL during the first two hours of heating (Table 1). With the extension of heat treatment time, the rate of protein degradation into peptides and amino acids increased, leading to a decrease in total protein content after 3 h. This is consistent with the research results of Zhang et al., in which the protein hydrolysis speed accelerates and the content of peptides increases at a certain temperature and time [28].

**Figure 3:**
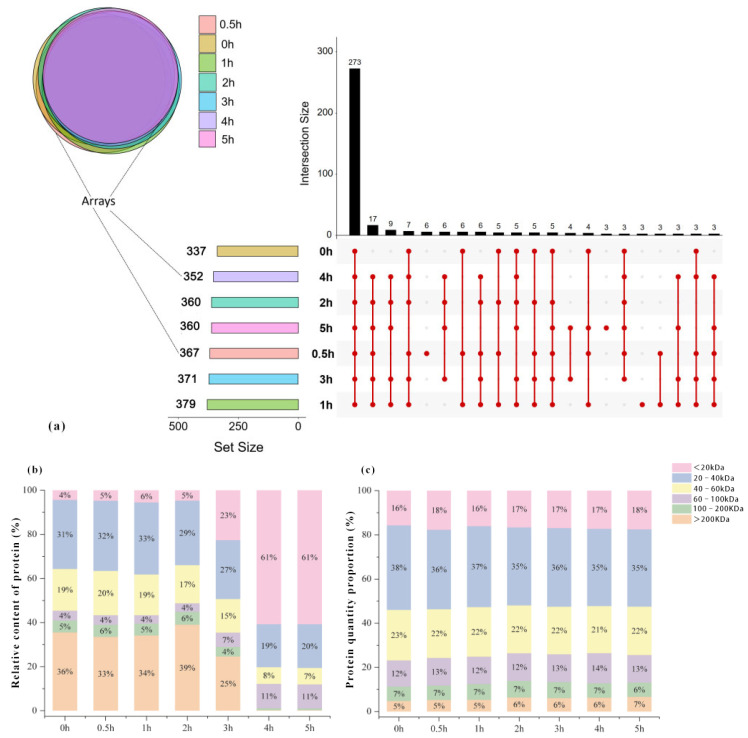
Protein kinds (**a**), relative content (**b**), and quantity proportion (**c**) in samples at different heat treatment times.

The protein analysis results of the samples with different heat treatment times are shown in Figure 3. A total of 422 proteins were identified in seven samples. Within 1 h of heat treatment, the number of proteins in the samples increased significantly from 337 (0 h) to 379. Although the 3 h heat-treated sample exhibited the lowest total protein quantity, its protein composition (371 proteins) showed no significant variation from the 1 h treatment group. Subsequent heat treatment between 4 and 5 h maintained comparable protein diversity (4 h: 352 proteins; 5 h: 360 proteins). The analysis of the number of proteins that overlap in chicken samples under different heat treatment times showed that the common protein shared by the seven groups of chicken samples was the largest (273 proteins), followed by the heat-treated groups (0.5–5 h, 17 proteins). Among the seven groups of samples, six proteins were unique to the 0.5 h sample (A0A1D5PI23, A0A1D5PMT8, A0A8V0Y0Y7, P18660, A0A8V0YQ99, P00789), followed by the 1 h heated sample (three kinds: A0A8V0XZA8, A0A8V0ZLU6, A0A8V1A1G2) and the 5 h heated sample (three kinds: A0A8V0ZGJ3, F1NMP5, Q5ZHW8).

**Figure 4:**
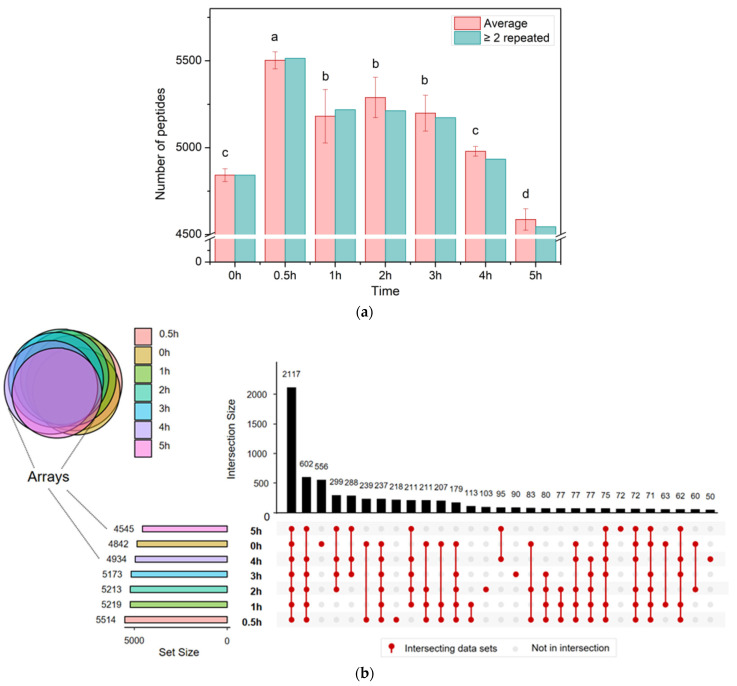
Peptide kinds in samples at different heat treatment times: (**a**) comparison of peptide numbers, significant differences are represented by different letters (*p* < 0.05); (**b**) peptide overlap in seven samples.

Proteins were categorized into six intervals based on molecular weight (Figure 4), including <20 kDa, 20−40 kDa, 60−100 kDa, 100−200 kDa, and >200 kDa. Quantitative analysis revealed a progressive decline in the >200 kDa fraction as heating time increased. The percentage of the >200 kDa fraction decreased rapidly from 39% (2 h) to 25% (3 h) after heating time exceeding 2 h, and decreased to 1% with a further extension of the heating time. The relative content of protein with a molecular weight of <20 kDa showed an overall increasing trend with the increase in heating time. The percentage of the <20 kDa fraction increased rapidly from 5% (2 h) to 23% (3 h) after heating time exceeding 2 h, and increased to 61% with a further extension of the heating time. The results showed that the increase in cooking time effectively promoted the degradation of proteins in chicken, and significantly increased the level of low molecular weight proteins, which is consistent with the findings of Qi et al. that prolonged simmering increased the concentration of small molecule components (<10 kDa) in chicken soup [29]. In contrast, the stewing process has less effect on the number of proteins with different molecular weights.

### 3.4. Peptidomics Characterization Analysis

The results of the determination of peptide content in chicken soup samples under different heat treatment times are shown in Table 1. Chicken breast is rich in water-soluble peptides, and high levels of peptides (26.70 mg/mL) are diffused from the raw meat into the water when the raw meat is left in water for a short time. During the heating process, the release of peptides in chicken meat was significantly accelerated in the initial heating stage (0–0.5 h), and the total amount of peptides in chicken soup showed an upward trend with the extension of time (0.5–4 h). When heating is extended to 5 h, the peptide content in chicken soup decreases, likely due to the utilization of some peptides as substrates (e.g., Maillard reaction), leading to greater consumption than production.

Figure 4 presents the peptide analysis results for samples subjected to different heat durations. A total of 8018 peptides were identified in chicken soup with heat treatment of 0–5 h. From Figure 4, the chicken breast was extremely rich in peptides (4842 ± 37 (0 h)), presumably related to the variety of enzymes in chicken tissues. During the storage of post-mortem chicken, these enzymes break down the proteins and release peptides, which provide abundant precursors for the Maillard reaction. During the initial heating stage (0–0.5 h), the number of peptide types (5503 ± 49 at 0.5 h) increased significantly due to protein denaturation caused by heat, indicating that the increased temperature promotes protein degradation and facilitates peptide formation. There was no significant difference in peptide types during 1–3 h of heat treatment (5181 ± 154 (1 h), 5289 ± 116 (2 h), 5199 ± 103 (3 h)). When the time exceeded 3 h, the types of peptides showed a significant decreasing trend (4979 ± 28 (4 h), 4587 ± 62 (5 h)), and it was speculated that the peptides in chicken soup would be aggregated by heat or consumed in the Maillard reaction as the extension of heat treatment time.

The analysis of peptide diversity in chicken soup samples under varying heat treatment durations revealed that the seven sample groups shared the most peptides, with 2117 types, followed by the heat-treated groups (0.5–5 h) with 602 types. Compared with the heat treatment group (0.5–5 h), the unheated sample (0 h) had the largest number of unique peptides (556 kinds). The 0.5 h sample contained 218 unique peptides, making it second only to the unheated sample. The number of unique peptides in the 1–5 h chicken soup samples remained relatively low (48–103 types) as heating increased.

### 3.5. Composition and Relative Abundance Analysis of Umami Peptides

With the in-depth study of the structure-activity relationship of umami peptides and the development of virtual screening technology, a variety of umami peptide prediction platforms have been developed recently and have shown excellent prediction potential, such as iUmami-SCM, UMPred-FRL, Umami_YYDS, and TastePertides-DM [30,31,32]. Four umami peptide prediction platforms were used to assess the umami of the identified peptides. The results showed that the number of umami peptides screened by TastePertides-DM and Umami_YYDS was higher at 7583 and 7525, respectively, compared to 4913 and 4004 peptides screened by UMPred-FRL and iUmami-SCM platforms. To enhance umami peptide prediction accuracy, the 2323 peptides were identified through joint screening across four platforms (iUmami-SCM, UMPred-FRL, Umami_YYDS, and TastePertides-DM).

In order to clarify the composition characteristics of umami peptides, they were analyzed from four aspects, including peptide sequence length, C-terminal/N-terminal amino acid composition, proportion of hydrophilic amino acids, and structure of continuous acidic amino acids. From Figure S1, the identified umami peptides predominantly consisted of 11–18 amino acid residues, with pentadecapeptides being the most prevalent (218 peptides). The identification of peptides is closely related to the instrumental conditions and peptide analysis methods, and the relationship between peptide chain length and umami needs to be further explored. Statistical analysis of terminal amino acids in umami peptides revealed distinct structural patterns. The N-terminal of the umami peptides was mainly composed of Ser, Ala, Glu, Thr, and Lys, and peptides with these five amino acids as N-terminal accounted for 52.48% of the total peptides; their adjacent positions were mostly Asp and Glu. The C-terminal analysis demonstrated the predominant occurrence of Lys, Arg, Ser, and Glu, which together accounted for 49.20% of total peptides. The adjacent positions of the C-terminal were mostly Ala, Leu, and Ser. The proportion of hydrophilic amino acid residues in umami peptides ranged mostly 50–80% and the types of amino acid residues at the C-terminal and N-terminal of the peptide are consistent with existing research and constitute a critical determinant for umami perception [33].

### 3.6. Fingerprint of Umami Peptides

The results of the protein sources of umami peptides (2323 peptides) are shown in Table S6. The Top 5 proteins were traced according to the types of protein degradation to produce umami peptides (Table 2), namely titin (A0A8V0ZZ81, 557 umami peptides), nebulin (A0A8V0XZQ1, 355 umami peptides), myosin heavy chain (P13538, 165 umami peptides), Myomesin-1 (F1N9Z6, 101 umami peptides), and LIM domain binding 3 (A0A8V0Y5G5, 93 umami peptides). Among them, the largest variety of umami peptides came from titin, accounting for 24% of the identified umami peptides, and the quantitative results showed that titin (A0A8V0ZZ81) showed a significant downward trend during stewing for 1–2 h. Further, the degradation characteristics of the Top 5 proteins and the effects of heat treatment on their peptide fingerprints were analyzed by Peptigram software. Draw the key cleavage sites of the Top 5 proteins using the online tool NovoPro (https://www.novopro.cn/tools/, accessed on 30 May 2025).

**Figure 5:**
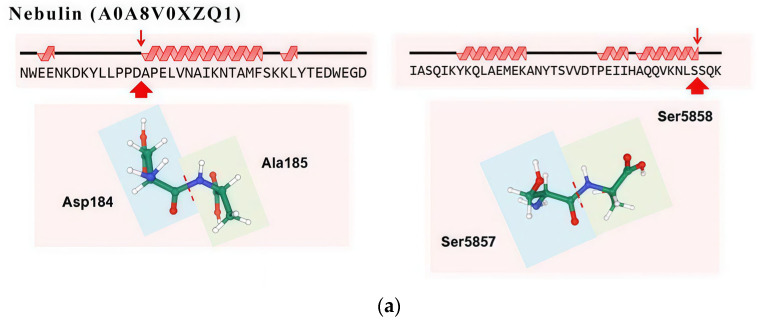

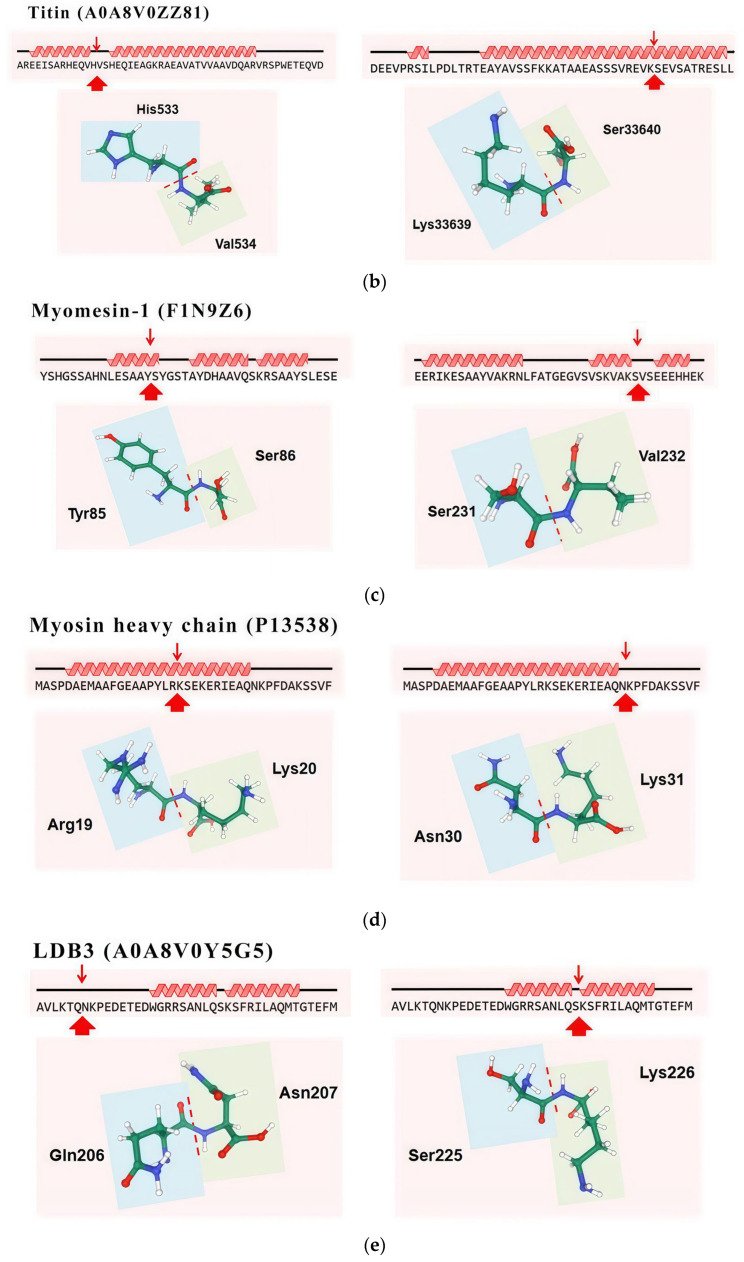
Key proteolytic cleavage site of titin (**a**), nebulin (**b**), myosin heavy chain (**c**), Myomesin-1 (**d**), and LDB3 (**e**).

Chicken breast is known to be a typical skeletal muscle, mainly composed of muscle bundles containing muscle fibers arranged in bundles [34]. Titin is a giant sarcomeric protein with complex molecular folding functions and is the third most abundant protein in skeletal muscle fibers [35]. Peptigram analysis of titin (A0A8V0ZZ81) revealed that proteolytic cleavage predominantly occurred between Val534 and His533, as well as between Lys33639 and Ser33640 (Figure 5a). Val534 and Lys33639 are the key N-terminal and C-terminal amino acids of umami peptides, respectively. Efficient cleavage of these sites is a prerequisite for the release of potentially umami peptides, thus establishing a direct and critical link to the umami properties of the final product. The predominant umami peptide SFKKATAAEASSSVREVK (SK18) demonstrated maximum abundance at 3 h of stewing, followed by progressive degradation with extended stewing time.

Nebulin is a giant actin-binding protein that regulates muscle contraction by influencing the binding of actin and myosin through interactions with troponin [36]. Umami peptides from nebulin (A0A8V0XZQ1) were predominantly generated through cleavage between residues 55–1375 (N-terminal) and 4900–6287 (C-terminal). Among them, the most abundant umami peptide SEKEYRKDLEEGVKGKG (SG17) reached its highest concentration in the soup stewed for 1 h, gradually decreasing as the stewing time increased. Ala185 and Ser5857 were the key N-terminal and C-terminal amino acids of the umami peptides derived from nebulin A0A8V0XZQ1. Key cleavage sites were identified between Asp184 and Ala185, as well as between Ser5857 and Ser5858 (Figure 5b).

Myosin heavy chain is a fundamental structural component of myosin and plays a crucial role in maintaining normal muscle cell function. Saneyasu et al. demonstrated that the myosin heavy chain gene is a critical factor in increasing chicken breast muscle weight during the first week post-hatching [37]. Umami peptides from myosin heavy chain (P13538) exhibited dispersed cleavage sites, primarily between N-terminal positions 3 and 97. The peptide KEVDVSIKGEAVREDHLLLR (KR20) peaked at 2–3 h, and its abundance gradually decreased with the extension of stewing time. Key cleavage sites were identified between Arg19 and Lys20, as well as Asn30 and Lys31 (Figure 5c).

Myomesin-1 is a protein encoded by the MYOM1 gene and is expressed in almost all striated muscle [38]. The analysis of the degradation characteristics of Myomesin-1 (F1N9Z6) revealed that its cleavage sites are relatively concentrated. The peptide SAAYSYGSTAYDHAAVQSKRSAAYS (SS25) reached its maximum abundance at 2 h, and its abundance gradually declined with extended stewing time. Ser86 and Ser231 are the key N-terminal and C-terminal amino acids of the umami peptide, respectively. The breakage of the peptide bond between Tyr85 and Ser86, as well as between Ser231 and Val232, facilitates umami peptide production (Figure 5d).

LIM domain binding 3 (LDB3) is a protein containing a PDZ domain that interacts with α-actinin-2 through its PDZ domain and is involved in the assembly of the cytoskeleton [39]. Studies have shown that, in addition to the smaller molecular polypeptides formed by irradiation of muscle LIM protein, other proteins such as titin, nebulin, troponin, desmin, and myotilin significantly affect the taste of the meat [40]. In the present work, LDB3 (A0A8V0Y5G5) generated umami peptides by cleaving the peptide bond between amino acid residues 201–231. The dominant peptide TQNKPEDETEDWGRRSANLQSKSFR (TR25) peaked at 2 h and its abundance gradually decreased with the extension of stewing time. Asn207 and Ser225 were the key N-terminal and C-terminal amino acid residues of the umami peptides derived from LDB3 protein (Figure 5e).

### 3.7. Release Patterns of Umami Peptides

**Figure 6:**
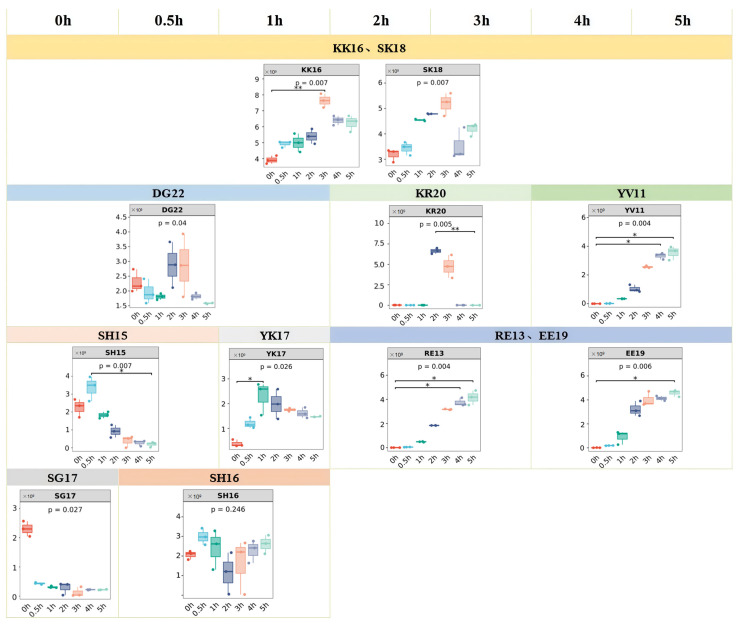
The release patterns of umami peptides with higher abundance (*: *p* < 0.05; **: *p* < 0.01).

The Top 5 umami peptides in the samples at different stewing times were counted according to their abundance ranking, and their release patterns were analyzed. Figure 6 illustrates peptide content dynamics across stewing times, plotted by the bioinformatics platform GenesCloud (https://www.genescloud.cn/). According to Table 3 and Figure 6, 11 highly abundant umami peptides were identified in samples stewed for 0–5 h: KKATAAEASSSVREVK (KK16), SFKKATAAEASSSVREVK (SK18), RVVDLMVHMASKE (RE13), EFGYSNRVVDLMVHMASKE (EE19), YEAFVKHIMSV (YV11), KEVDVSIKGEAVREDHLLLR (KR20), DWRKNIEEKSGMEGRKKMFEAG (DG22), SSPHQHDQEVKSHALH (SH16), YEHHASSEEKITASEEK (YK17), SPHQHDQEVKSHALH (SH15), and SEKEYRKDLEEGVKGKG (SG17). Among these peptides, KK16 and SK18, which were derived from different types of titin and both terminate with a conserved C-terminal lysine residue, were ranked among the Top 5 in terms of abundance in 0–5 h stewing samples. It is hypothesized that these peptides significantly contribute to the overall taste of chicken soup. The abundance analysis revealed that both peptides (KK16 and SK18) peaked in the samples stewed for 3 h, and their levels demonstrated an initial increase followed by a gradual decline as the stewing time extended. Umami peptides SH15, KR20, and YK17 exhibited maximum abundance in samples stewed for 0–0.5 h, 2–3 h, and 1 h, respectively, and their abundances with initial increases followed by progressive declines as stewing time extended. The umami peptide SG17 reached peak abundance in samples with 0 h stewing duration, demonstrating an inversely proportional relationship between its concentration and stewing time. The abundance of two umami peptides, DG22 and SH16, was higher in samples stewed for 0–1 h and 0.5–1 h, respectively, and their abundance fluctuated throughout the stewing period. Umami peptides RE13, EE19, and YV11 exhibited peak abundance in samples stewed for 2–5 h, 2–5 h, and 4–5 h, respectively, with their abundance positively correlated to stewing time. These three peptides (RE13, EE19, and YV11) may serve as potential biomarkers for monitoring umami quality development during chicken soup stewing, as their accumulation aligns with the progressive enhancement of umami intensity.

## 4. Conclusions

In summary, the protein degradation and umami peptide release patterns in stewed chicken were studied in this work. First, the temperature rise profile in the stewing pot during chicken soup preparation was monitored using a wireless temperature recorder, identifying seven sampling points. Additionally, the taste characteristics and peptide composition of chicken soup, as well as the protein composition of chicken meat at the seven sampling points, were analyzed. The results revealed that the molecular weights of proteins in chicken meat decreased significantly as the stewing time increased. Traceability analysis revealed that umami peptides were primarily derived from the degradation of titin, with Val534 and Lys33639 serving as the major N- and C-terminal amino acid residues. The obtained results should contribute to a better understanding of the unique taste of chicken soup. In the future, the development of targeted enrichment technology for umami peptides in chicken soup will be helpful in promoting the production of high-quality chicken soup products.

## Figures and Tables

**Table 1:** The total content of protein and peptide in chicken soup.

	Heat Treatment Time						
	0 h	0.5 h	1 h	2 h	3 h	4 h	5 h
Protein content (mg/mL)	34.95 ± 1.13 ^c^	37.52 ± 1.18 ^c^	61.39 ± 1.01 ^a^	62.68 ± 1.37 ^a^	37.03 ± 1.41 ^c^	52.48 ± 1.96 ^b^	60.40 ± 2.34 ^a^
Peptide content (mg/mL)	26.70 ± 1.68 ^f^	47.10 ± 1.57 ^e^	52.50 ± 1.11 ^d^	56.60 ± 1.47 ^c^	57.60 ± 2.18 ^c^	75.00 ± 1.50 ^a^	66.30 ± 2.07 ^b^

^a~f^ Significant differences are represented by different letters (*p* < 0.05).

**Table 2:** Umami peptides traced back to Top 5 proteins.

	Accession Number	Peptide Length	Molecular Weight	Protein Name	Peptides Quantity
1	A0A8V0ZZ81	34,378	3,820,161	Titin	557
2	A0A8V0XZQ1	6356	735,970	Nebulin	355
3	P13538	1939	223,145	Myosin heavy chain	165
4	F1N9Z6	1587	177,328	Myomesin-1	101
5	A0A8V0Y5G5	701	75,370	LIM domain binding 3	93

**Table 3:** Summary of the Top 5 umami peptides with the highest abundance in samples at different heat treatment times.

No.	Peptide Sequence	Abbreviations	Protein Source
1	KKATAAEASSSVREVK	KK16	A0A8V0ZUU8
2	SFKKATAAEASSSVREVK	SK18	A0A8V0ZZ81
3	RVVDLMVHMASKE	RE13	A0A8V0ZZ81
4	EFGYSNRVVDLMVHMASKE	EE19	A0A8V0ZZ14
5	YEAFVKHIMSV	YV11	P68246
6	KEVDVSIKGEAVREDHLLLR	KR20	P13538
7	DWRKNIEEKSGMEGRKKMFEAG	DG22	A0A8V0X091
8	SSPHQHDQEVKSHALH	SH16	A0A8V0Z679
9	YEHHASSEEKITASEEK	YK17	A0A8V0ZZ81
10	SPHQHDQEVKSHALH	SH15	A0A8V0XGP3
11	SEKEYRKDLEEGVKGKG	SG17	A0A8V0XZQ1

## Data Availability

The original contributions presented in the study are included in the article, further inquiries can be directed to the corresponding author.

## Supplementary Materials

The following supporting information can be downloaded at: https://www.mdpi.com/article/10.3390/foods14142497/s1, Figure S1: Composition characteristics of umami peptides (Peptide sequence length (a), C-terminal/N-terminal amino acid composition (b), proportion of hydrophilic amino acids (c), structure of continuous acidic amino acids (d)); Table S1: GL1_test procedure; Table S2: Sample_Measurement (2steps_washing) procedure; Table S3: Gradient elution procedure; Table S4: Mass spectrometry parameters; Table S5: Search the database parameters; Table S6: Source protein distribution of umami peptides.
