# Supplementary material for: Analysis of Protein Degradation and Umami Peptide Release Patterns in Stewed Chicken Based on Proteomics Combined with Peptidomics Approach
Source: Foods. 2025 Jul 16;14(14):2497. doi: 10.3390/foods14142497 (PMC12294777; doi:10.3390/foods14142497)
Supplement: Supplementary file 1 [file foods-14-02497-s001.zip › foods-3704682-supplementary.pdf]

Supplementary information

Figure S1. Composition characteristics of umami peptides (Peptide sequence length (**a**), C-terminal/N-terminal amino acid composition (**b**), proportion of hydrophilic amino acids (**c**), structure of continuous acidic amino acids (**d**)).

Table S1. GL1_test procedure.

Table S2. Sample_Measurement (2steps_washing) procedure.

Table S3. Gradient elution procedure.

Table S4. Mass spectrometry parameters.

Table S5. Search the database parameters.

Table S6. Source protein distribution of umami peptides.

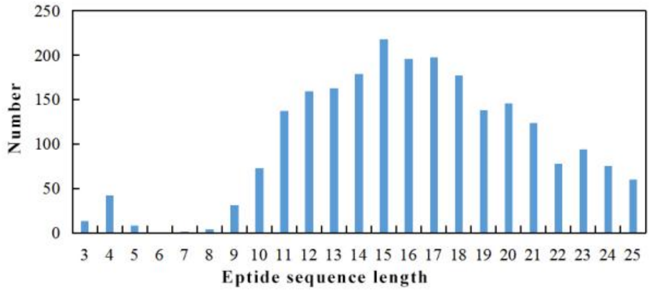

(**a**)

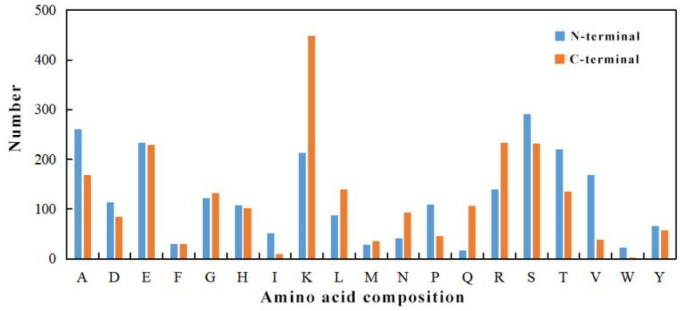

(**b**)

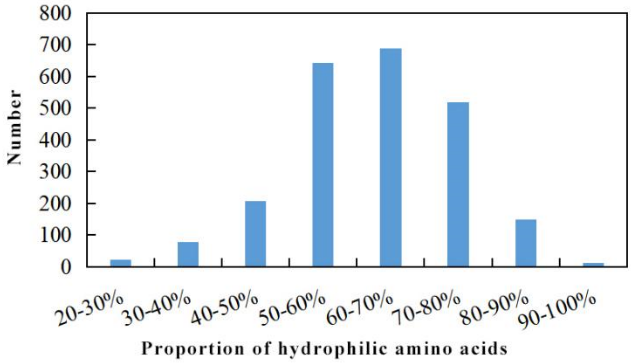

(**c**)

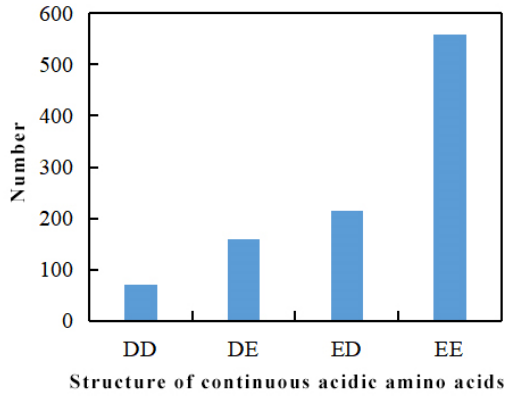

(**d**)

Figure S1. Composition characteristics of umami peptides (peptide sequence length (**a**), C-terminal/N-terminal amino acid composition (**b**), proportion of hydrophilic amino acids (**c**), structure of continuous acidic amino acids (**d**)).

Table S1. GL1_test procedure

Solution	Measurement time (s)	Rinsing time (s)	Cut-off criteria	Stability threshold (mV)							
				1ch	2ch	3ch	4ch	5ch	6ch	7ch	8ch
Cleaning 1	-	90	-	-	-	-	-	-	-	-	-
Cleaning 2	-	120	-	-	-	-	-	-	-	-	-
Cleaning 3	-	120	-	-	-	-	-	-	-	-	-
Calibration	30	-	1	0.5	0.5	0.5	0.5	100	0.5	0.5	0.5
Sample	30	-	-	-	-	-	-	-	-	-	-
Post-rinse 4	-	3	-	-	-	-	-	-	-	-	-
Post-rinse 5	-	3	-	-	-	-	-	-		-	-
CPA Solution	30	-	-	-	-	-	-	-	-	-	-

Table S2. Sample_Measurement (2steps_washing) procedure

Solution	Measurement time (s)	Rinsing time (s)	Cut-off criteria	Stability threshold (mV)							
				1ch	2ch	3ch	4ch	5ch	6ch	7ch	8ch
Cleaning 1	-	90	-	-	-	-	-	-	-	-	-
Cleaning 2	-	120	-	-	-	-	-	-	-	-	-
Cleaning 3	-	120	-	-	-	-	-	-	-	-	-
Calibration	30	-	20	0.5	0.5	0.5	0.5	0.5	0.5	0.5	0.5
Sample	30	-	-	-	-	-	-	-	-	-	-
Post-rinse 4	-	3	-	-	-	-	-	-	-	-	-
Post-rinse 5	-	3	-	-	-	-	-	-		-	-
CPA Solution	30	-	-	-	-	-	-	-	-	-	-

Table S3. Gradient elution procedure

Time (min)	Proportion of mobile phase B (%)
0-50	0-35
50-58	35-100
58-60	100

Table S4. Mass spectrometry parameters

Parameter	Setting
MS1 Scan Range	300-1800 *m/z*
MS1 Resolution	70,000 (@ *m/z* 200)
Automatic Gain Control	$3e^6$
Maximum Injection Time	10 ms
Dynamic Exclusion Duration	40.0 s
MS2 Fragmentation Mode	HCD
Isolation Window	2 *m/z*
MS2 Resolution	17,500 (@ *m/z* 200)
Normalized Collision Energy	30 eV
Underfill ratio	0.1 %

Table S5. Search the database parameters

Parameter	Setting
Main search ppm	6
Max Missed Cleavages	2
De-Isotopic	True
Enzyme	Trypsin (Protein)/Unspecific (Peptide)
Fixed modifications	Carbamidomethyl (C)
Variable modifications	Oxidation (M)
Database	uniprot_Gallus_gallus_43710_20230213
Lable free quantification (LFQ)	True
LFQ min ratio count	1
Match between runs	2 min
Peptide Mass Tolerance	± 20ppm
Petide FDR	≤ 0.01
Protein FDR	≤ 0.01

Table S6. Source protein distribution of umami peptides

No.	Protein	Peptides quantity	No.	Protein	Peptides quantity	No.	Protein	Peptides quantity
1	A0A8V0ZZ81	557	50	P12902	6	99	Q5ZM35	2
2	A0A8V0XZQ1	355	51	Q9PSW9	6	100	Q90705	2
3	P13538	165	52	A0A8V0XH44	5	101	A0A1D5PHJ8	1
4	F1N9Z6	101	53	A0A8V0ZZ14	5	102	A0A1L1RZK1	1
5	A0A8V0Y5G5	93	54	A0A8V1A558	5	103	A0A3Q3AHU9	1
6	P68246	85	55	A0A8V1A999	5	104	A0A8V0X3T8	1
7	A0A8V0ZUU8	55	56	A0A8V1ALZ7	5	105	A0A8V0XEH2	1
8	P00565	44	57	Q5ZIR5	5	106	A0A8V0XLY1	1
9	A0A8V0X4F7	38	58	Q5ZK95	5	107	A0A8V0XN75	1
10	A0A8V0ZIK7	37	59	R4GJM5	5	108	A0A8V0XXW6	1
11	A0A8V0XBW5	27	60	A0A1L1RQ67	4	109	A0A8V0XZJ1	1
12	P02605	27	61	A0A8V0XCG0	4	110	A0A8V0Y807	1
13	A0A8V0XAE4	26	62	A0A8V0YM75	4	111	A0A8V0YE67	1
14	A0A8V0XGP3	26	63	A0A8V0YQ99	4	112	A0A8V0YMT7	1
15	A0A8V0YVG5	26	64	A0A8V0YZ04	4	113	A0A8V0YNI4	1
16	A0A8V1A1P8	25	65	A0A8V0ZRJ3	4	114	A0A8V0YNS1	1
17	A0A8V0Z679	24	66	A0A8V1A8B6	4	115	A0A8V0YUF9	1
18	A0A8V0Z6R2	23	67	P00340	4	116	A0A8V0ZAM3	1
19	A0A8V1A5I9	23	68	P01994	4	117	A0A8V0ZCB2	1
20	P05081	20	69	P13585	4	118	A0A8V0ZHA8	1
21	P16419	20	70	A0A3Q2U3U3	3	119	A0A8V0ZI62	1
22	A0A8V0XM57	19	71	A0A8V0XYF4	3	120	A0A8V0ZIF8	1
23	P02609	18	72	A0A8V0Y8G8	3	121	A0A8V0ZMB3	1
24	A0A8V0Y463	17	73	A0A8V0YPY7	3	122	A0A8V0ZSH6	1
25	A0A8V1A3L9	17	74	A0A8V0Z6P9	3	123	A0A8V0ZT94	1
26	A0A8V0X091	16	75	A0A8V0ZW85	3	124	A0A8V0ZTA5	1
27	A0A8V0X9F2	16	76	A0A8V1AEW4	3	125	A0A8V0ZZ38	1
28	A0A8V0Y9B6	16	77	F1NN63	3	126	A0A8V1A1B3	1
29	A0A8V0YFE1	13	78	P19966	3	127	A0A8V1A3F1	1
30	A0A8V0ZH41	13	79	A0A1L1RIX9	2	128	A0A8V1A4H7	1
31	P68139	13	80	A0A8V0XEY6	2	129	A0A8V1A4Q9	1
32	Q5ZLN1	13	81	A0A8V0XF24	2	130	A0A8V1A6Y9	1
33	A0A8V0XDS3	12	82	A0A8V0Y599	2	131	A0A8V1A8S7	1
34	F1NZ04	12	83	A0A8V0Y7L7	2	132	A0A8V1ACU4	1
35	P00356	12	84	A0A8V0YJQ0	2	133	A0A8V1AH75	1
36	F1N9H4	11	85	A0A8V0YVM4	2	134	A0A8V1AJR9	1
37	P07322	11	86	A0A8V0ZIB3	2	135	A0A8V1AKS6	1
38	A0A8V1AKP7	10	87	A0A8V0ZLU6	2	136	A0A8V1APN3	1
39	A0A8V1A4K7	9	88	A0A8V0ZT61	2	137	E1C3A9	1
40	Q90577	9	89	A0A8V1A1X3	2	138	P00504	1
41	A0A3Q3ANR7	8	90	A0A8V1A2K7	2	139	P00508	1
42	A0A8V1A0S8	8	91	A0A8V1A9G6	2	140	P09860	1
43	A0A8V1AK68	7	92	A0A8V1ACX4	2	141	P0CB50	1
44	Q05623	7	93	P00548	2	142	P11009	1
45	A0A1D5PMT8	6	94	P00940	2	143	Q00649	1
46	A0A8V0Z7V9	6	95	P02001	2	144	Q5ZLC5	1
47	E1BQD1	6	96	P02314	2	145	Q90835	1
48	P02197	6	97	P02588	2	146	Q9PRL8	1
49	P04268	6	98	P11501	2			
